# VirDiG: a *de novo* transcriptome assembler for coronavirus

**DOI:** 10.1093/bioadv/vbaf075

**Published:** 2025-04-08

**Authors:** Minghao Li, Xuaoyu Guo, Jin Zhao

**Affiliations:** School of Computer Science and Technology, Qingdao University, Shandong 266071, China; School of Computer Science and Technology, Qingdao University, Shandong 266071, China; School of Computer Science and Technology, Qingdao University, Shandong 266071, China

## Abstract

**Motivation:**

The discontinuous transcription mechanism of coronaviruses contributes to their adaptation to different host environments and plays a critical role in their lifecycle. Accurate assembly of coronavirus transcripts is vital for understanding the virus’s biological traits and developing precise prevention and treatment strategies. However, existing *de novo* assembly algorithms are primarily designed for alternative splicing events in eukaryotes and are not suitable for assembling coronavirus transcriptome, which consists of both genomic RNA and subgenomic mRNAs. Coronavirus transcriptome reconstruction from short reads remains a challenging problem.

**Results:**

In this work, we present VirDiG, a *de novo* transcriptome assembler specifically designed for coronaviruses. VirDiG utilizes a discontinuous graph to facilitate accurate transcript assembly by incorporating information from paired-end reads, sequence depth, and start and stop codons. Experimental results from both simulated and real datasets show that VirDiG exhibits significant advantages in reconstructing the transcriptome of coronaviruses when compared to traditional *de novo* assemblers tailored for classical eukaryotic transcriptome assembly.

**Availability and implementation:**

VirDiG is freely available at https://github.com/Limh616/VirDiG.git.

## 1 Introduction

**Figure 1.**
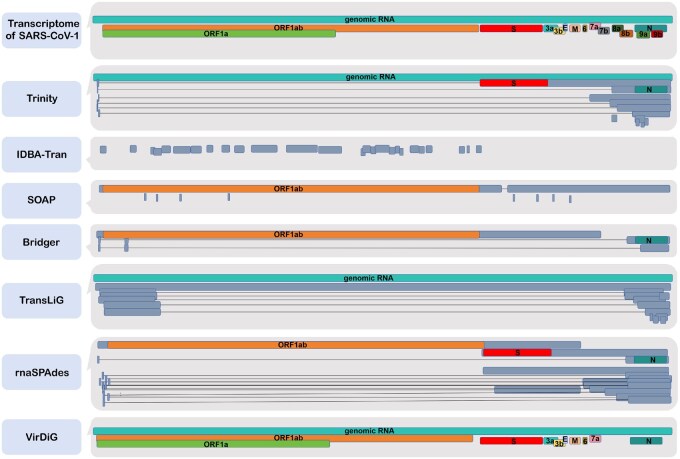
A detailed comparison between the authentic SARS-CoV-1 transcriptome and the transcript candidates generated by *de novo* assemblers using the SRR1942954 dataset. Coloured contigs indicate accurate transcript regions, while grey contigs represent non-transcript regions. The text within the coloured contigs corresponds to the transcript ID.

Coronaviruses possess a linear, single-stranded, positive-sense RNA genome. The expression of genes in coronaviruses is accomplished through a distinct process called discontinuous transcription, managed by the viral RNA-dependent RNA polymerase (RdRp) (Malone *et al.* 2022, Wells *et al.* 2023). In this unique transcription process, transcripts usually contain a 5’ leader sequence, which joins with the primary body sequence of the genome to construct the complete transcript sequence (Kim *et al.* 2020). Specifically, RdRp can skip over contiguous genomic regions or segments in the viral RNA template. This leads to a repertoire of discontinuous transcripts that correspond to specific subsequences of segments ordered as in the reference genome (Finkel *et al.* 2021). This process is distinct from the alternative splicing observed in eukaryotes, where a single gene can produce multiple mRNAs with varying combinations of exons (Stamm *et al.* 2005, Tazi *et al.* 2009). The coronavirus transcriptome consistently contains a genomic RNA, i.e. the complete viral genome without any skips or jumps. Therefore, the mRNAs of coronaviruses usually appear as subsequences of the genomic RNA, as illustrated in Fig. 1. This greatly complicates the assembly of coronavirus transcriptome.

A number of transcriptome assemblers have been developed in the past few years, and they can be divided into two broad categories: reference-based and *de novo* assemblers (Meleshko *et al.* 2021, Shumate *et al.* 2022). The primary distinction between these two strategies is that the former require reference genome, whereas the latter do not have this requirement. The reference-based assemblers usually achieve high accuracy with the support of a high-quality reference genome (Trapnell *et al.* 2012, Shao and Kingsford 2017). However, *de novo* assemblers are necessary when the reference genome is unavailable, incomplete, or inaccurate, particularly for coronaviruses with exceptionally high mutation rates (Robertson *et al.* 2010, Martin and Wang 2011). In this paper, we focus on developing a *de novo* transcriptome assembler for coronaviruses.

Existing *de novo* assemblers such as TransLiG (Liu *et al.* 2019), rnaSPAdes (Bushmanova *et al.* 2019), BinPacker (Liu *et al.* 2016), Bridger (Chang *et al.* 2015), Trinity (Grabherr *et al.* 2011), SOAPdenovo-trans (Xie *et al.* 2014), and IDBA-Tran (Peng *et al.* 2013) are specifically designed for eukaryotes. These assemblers usually first construct splicing graphs (Pertea *et al.* 2015, Zhao *et al.* 2022) or de Bruijn graphs (Zerbino and Birney 2008, Bankevich *et al.* 2012) to depict alternative splicing events, and then extract paths from these graphs to represent transcripts. The transcriptome of coronaviruses typically comprises a long genomic RNA that spans the entire genome and several shorter mRNAs (containing just one or multiple segments of the genome) resulting from discontinuous transcription. In the splicing graph or de Bruijn graph constructed by the above algorithms, the transcriptome of coronaviruses often corresponds to a long path, accompanied by shorter subpaths containing only one or several vertices along with this main path. However, the paths obtained by the above assemblers often incorporate fragments from multiple genuine transcripts, a challenge termed the ‘*Multiple Fragment Fusion Problem*’ in this paper. Besides, the graph models constructed by the aforementioned algorithms often struggle to capture all the discontinuous transcription events in coronaviruses. Figure 1 illustrates the outcomes of existing algorithms in assembling SARS-CoV-1 transcriptome with real dataset SRR1942954, indicating that their accuracy falls below practical requirements. Therefore, the classical transcript assemblers are inadequate for addressing the assembly challenge of coronavirus. An effective assembly algorithm for coronavirus transcriptome should fully account for the fact that coronaviruses possess a genomic RNA covering the entire genome, with many mRNAs being subsequences of this RNA.

The Jumper (Sashittal *et al.* 2021) and Cov-trans (Guo *et al.* 2024) algorithms are specifically designed for assembling coronavirus transcripts. Both use a reference-based assembly strategy with three key steps. First, they align RNA-seq reads to the reference genome using a splice-aware aligner (Kim *et al.* 2015, 2019). Next, they construct a graph model based on these alignments. Finally, they extract paths from the graph to represent transcripts. Notably, Cov-trans utilizes the start codon information to correct the graph’s boundaries, allowing it to assemble more transcripts with highly accurate boundaries. While Cov-trans achieves high accuracy, it requires a high-quality reference genome. It is not applicable when the genome is unknown, is gapped, highly fragmented or substantially altered. Therefore, a *de novo* transcriptome assembly algorithm specifically designed for coronaviruses is necessary.

In this study, we introduce a new *de novo* assembler called VirDiG, which effectively addresses the ‘*Multiple Fragment Fusion Problem*’ that existed in previous assemblers. VirDiG introduces a novel graph model to depict discontinuous transcription events. It employs a carefully designed approach to extract pathways for transcript recovery, considering factors such as sequencing depth, paired-end details, and start and stop codon information. Experimental results indicate that VirDiG outperforms the state-of-the-art assemblers Trinity, Bridger, BinPacker, TransLiG, rnaSPAdes, IDBA-Tran, and SOAPdenovo-trans on both simulated and real datasets.

## 2 Methods

### 2.1 Data collection

In this study, we gathered 3 simulated datasets and 9 real datasets from SARS-CoV-1, MERS-CoV, and SARS-CoV-2 for benchmarking.

- Simulated datasets. The simulated datasets were generated using Flux Simulator tool (Griebel *et al.* 2012) with annotated transcriptomes of three well-known viruses: SARS-CoV-1, MERS-CoV and SARS-CoV-2.
- Real datasets. All real datasets were retrieved from the NCBI Sequence Read Archive database. Specifically, there are three samples with accession code SRR1942956, SRR1942957, and SRR1942954 for SARS-CoV-1. Samples with accession codes SRR10357372, SRR10357373, and SRR10357374 are from MERS-CoV. SARS-CoV-2 datasets consist of samples with accession codes SRR12789544, SRR12789557, and SRR12789558.

### 2.2 Contigs assembly and genomic RNA identification

VirDiG decomposes each read into overlapping k-mers, which are short subsequences of length k (typically set to 31 by default). It stores these k-mers in a hash table and records their occurrences. VirDiG employs the rules introduced in (Chang *et al.* 2015) to detect and remove probable sequencing errors. This involves comparing the frequency of k-mers within groups of similar k-1 prefixes or suffixes, ensuring that only the more reliable k-mers are retained. VirDiG uses the following strategy to construct contiguous sequences from fragmented k-mer data.

**Step 1.** Initial k-mer selection. At the beginning of each iteration, VirDiG selects an unused k-mer with the highest abundance and Shannon’s entropy greater than 1.5 to serve as the initial sequence for assembly.
**Step 2.** Sequence extension. The initial k-mer sequence is extended iteratively by continually selecting unused k-mers. These k-mers are chosen based on their highest abundance and must overlap by k−1 base pairs with either the 5’ end or the 3’ end of the current sequence.
**Step 3.** Contig formation. The extension process proceeds until there are no additional k-mers that can be used to further extend the sequence. At this point, the sequence is considered complete and forms a contiguous segment, referred to as a contig.
**Step 4.** Removing short contigs. VirDiG removes shorter contigs that may be generated due to erroneous k-mers. Additionally, it removes inverse complementary contigs that are contained within other contigs.
**Step 5.** Contigs merging. When the tail of one contig overlaps with the head of another contig, they are spliced together to form a longer contig. The merging process involves linking the tail of one contig with the head of another contig if they share a common subsequence. This iterative merging continues until no further mergers are possible.

VirDiG selects the longest contig and checks if it meets the following conditions:

- The number of k-mers in this contig surpasses 90% of all used k-mers.
- Its length is 20 times greater than the length of the second longest contig.
- Its length is 5 times the combined lengths than the combined lengths of the remaining contigs.

If any of these conditions are met, the longest contig is considered as genomic RNA, and a graph is constructed to represent the remaining mRNAs. Otherwise, mRNAs are identified from all the contigs according to codon information.

### 2.3 Construction of discontinuous graph

According to the transcription characteristics of coronaviruses, we proposed a more accurate graph model called the discontinuous graph. The discontinuous graph is initialized as a graph containing only one vertex, which stores the constructed genomic RNA. Subsequently, we employ the following strategy to partition the graph.

We label the genomic RNA as vertex $v_{1}$ and scan the sequence in vertex $v_{1}$ to find all instances of the start codon (‘*ATG*’). From these positions, we extract *k*-mers, where each *k*-mer starts with the start codon. We then map reads containing these *k*-mers to vertex $v_{1}$. Once the 5’ part of a read aligns with the 5’ leader sequence of the genomic RNA and the rest of the read jumps to align with the start codon, the read is called a jump read. If a significant number of jump reads are detected at the start codon, we split vertex $v_{1}$ at the start codon into two vertices, $v_{1}$ and $v_{2}$. Vertex $v_{2}$ represents the region from the 5’ end to just before the start codon, and vertex $v_{1}$ is updated to represent the region from the start codon to the 3’ end of the original $v_{1}$. Additionally, we introduce vertex $v_{3}$ to represent the portion of the beginning of the genomic RNA that maps to the jump reads, and add directed edges $v_{2}\to v_{1}$ and $v_{3}\to v_{1}$. Figure S1 in the appendix provides a detailed example.

Through the aforementioned strategy, we have completed the construction of a discontinuous graph. In this graph, the vertices that arise from genomic RNA are referred to as trunk vertices, whereas the vertices introduced through the incorporation of jump reads are designated as branch vertices. We then align all sequencing reads to the graph and assign weights to vertices. For each vertex v in the graph, its weight cov(v) corresponds to the average depth of reads aligned to its sequence.

The discontinuous graph utilized in this study differs from commonly used splicing graphs or de Bruijn graphs. During the construction of the discontinuous graph, the unique characteristic of discontinuous transcription in coronaviruses was taken into consideration. Instead of using individual k-mers, the original reads were directly employed in this strategy, which greatly reduces the number of erroneous edges, enabling the recovery of a greater number of true positives from a minimal set of candidate transcripts.

### 2.4 Recovery of transcripts

Theoretically, each transcript corresponds to a path in the discontinuous graph consisting of one branch vertex and multiple trunk vertices. In this representation, the branch vertex represents the leader sequence of a transcript, while trunk vertices represent the body of this transcript. Note that each vertex in the graph has at most one outgoing edge. This unique graph structure simplifies the extraction of transcript paths. We just need to start from each branch vertex and select a trunk vertex as the end point of the path. However, in practical applications, accurately identifying the endpoint can be challenging, and the transcript usually corresponds to a subsequence of the extracted path. To tackle this obstacle, we first utilize Algorithm 1 to extract candidate paths based on sequencing depth and paired-end information, and then refine these paths by considering the presence of start and stop codons.

Algorithm 1 **Input:** discontinuous graph G=(V,E)

**Output:** a set of path candidates P

1: P←∅

2: **for** each branch vertex $v_{0}$ in V  **do**

3:  $p\leftarrow \{v_{0}\}$

4:  $v\leftarrow v_{0}$

5:  **while**  $\text{score}(p,v)>C_{\text{min}}$  **do**

6:   p∪{v}

7:   **if**  v→child=NULL  **then**

8:    break

9:   **end if**

10:   v=v→child

11:  **end while**

12:  **if** number of vertices in p>1  **then**

13:   P=P∪{p}

14:  **end if**

15: **end for**

As described in Algorithm 1, we take each branch vertex as the starting vertex for a candidate path and iteratively extend the path by selecting vertices that satisfy score(p,v) > *C*_min_.

(1) $$\text{score}(p,v)=m+\left(-\text{log}\;\left|1-\frac{\text{cov}(v)}{\text{cov}(p)}\right|\right)$$

where,

(2) $$m=\{\begin{array}{cc} w, & \text{if}\;\text{map}(v_{0},v)>1\\ 0, & \text{else}\; \end{array}$$

 

(3) $$\text{cov}(p)=\sum_{v\in p}c\text{ov}(v)\times \frac{\text{len}(v)}{\sum_{u\in p}l\text{en}(u)}$$

where cov(v) and len(v) specify the average depth and length of the sequence associated with vertex v, respectively. $\text{map}(v_{0},v)$ denotes the number of paired-end reads that spanning both vertex $v_{0}$ and v. Additionally, the parameter w is an experimental value set to 0.7 by default.

For each candidate path or contig assembled in the previous step that is considered eligible as genomic RNA, we examine its sequence to pinpoint the positions of the start and stop codons. Subsequently, we determine the region between these codons as the candidate transcript for output. It is important to highlight that any duplicate transcripts are excluded from the output to maintain precision.

## 3 Results

**Figure 2.**
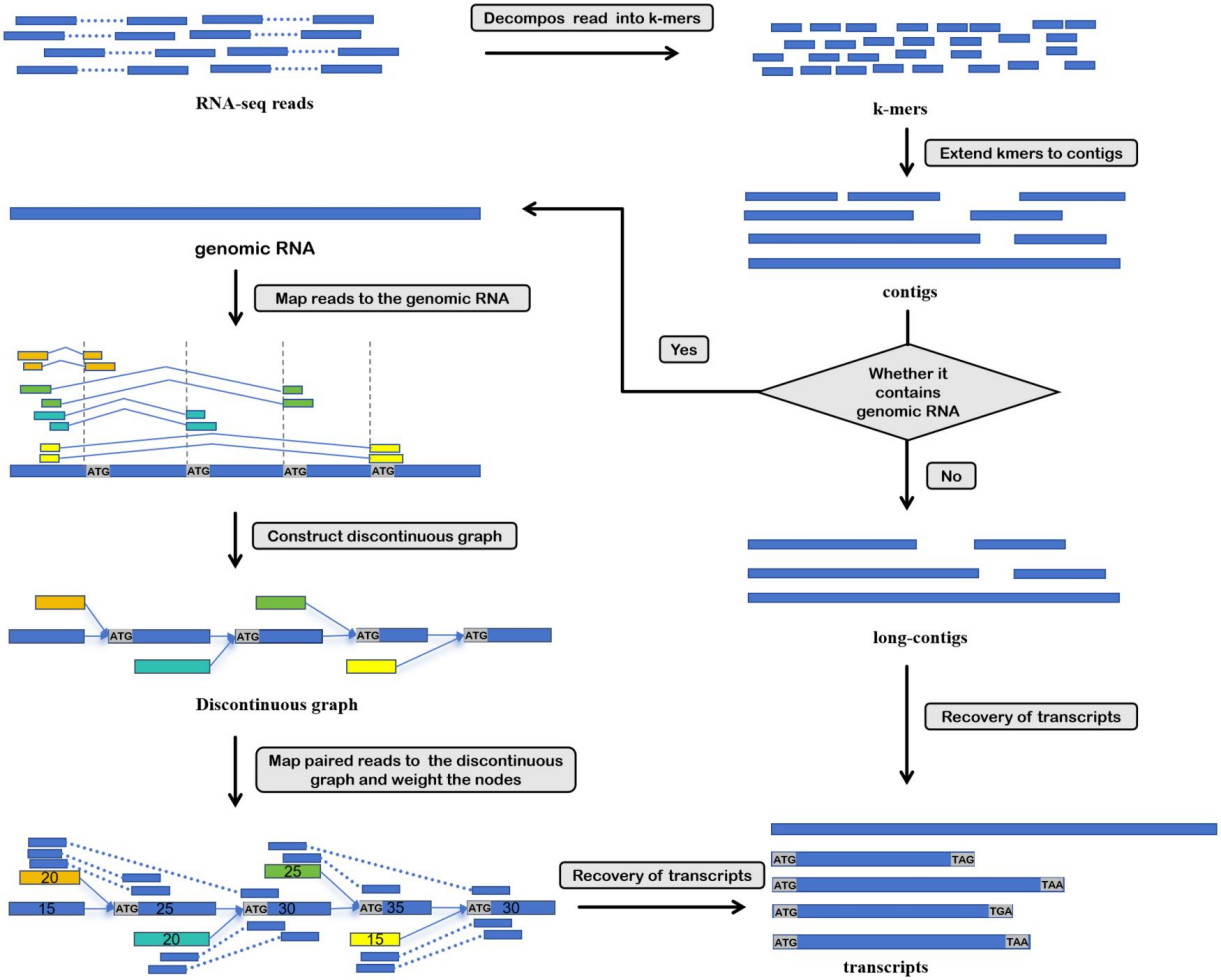
The workflow of VirDiG. The algorithm takes the paired-end reads as input. Initially, the reads are decomposed into k-mers, which are then extended into contigs. If the genomic RNA is present in these contigs, a discontinuous graph is constructed based on the alignment of reads at the start codon. VirDiG uses a greedy algorithm to reconstruct the transcript set, incorporating vertex abundance, paired-end reads, and codon information. If genomic RNA is absent, transcripts are identified from all the contigs according to codon information.

VirDiG is a *de novo* transcriptome assembler specifically designed for coronaviruses. VirDiG decomposes RNA-seq reads into k-mers and uses a greedy k-mer–based approach for fast and efficient contigs assembly. These contigs are then connected to form as long contigs as possible according to their overlap. VirDiG employs a strict criterion to assess if the longest contig represents the genomic RNA. If confirmed, reads containing the start codon are aligned to this contig to construct a directed acyclic graph, called discontinuous graph. Subsequently, paired-end reads are mapped to the vertexes of the discontinuous graph, assigning weights to all vertexes. Transcripts are then recovered according to the vertex weights, pair-end reads mapping locations, as well as the presence of start and stop codons. Besides, contigs that are not representative of genomic RNA are spliced into transcripts according to their length, coverage, and codon content. Note that the duplicate transcripts will be removed. The flowchart of the VirDiG is roughly outlined in Fig. 2.

### 3.1 Assessment metrics

We conducted benchmark tests comparing VirDiG with seven state-of-the-art *de novo* assemblers: rnaSPAdes, TransLiG, BinPacker, Bridger, Trinity, IDBA-Tran, and SOAPdenovo-trans on both simulated and real datasets. The assessment metrics used in the comparison are as follows:

Transcripts assembled by each algorithm were aligned to the ground truth transcripts using BLAT (Kent 2002) with 95% sequence identity as cutoff. A ground truth transcript is considered full-length reconstructed if it is covered by an assembled transcript with at least 95% sequence identity and no more than 5% indels. We benchmark the performances of assemblers using sensitivity, precision, and F1-score metrics. Sensitivity is calculated as the ratio of fully reconstructed transcripts to all expressed transcripts in the dataset. The precision is determined by the fraction of full-length reconstructed transcripts among all assembled transcripts. The F1 score takes into account both sensitivity and accuracy, which offers a more comprehensive assessment of the quality of the assembly.

### 3.2 Evaluation on simulated datasets

**Figure 3.**
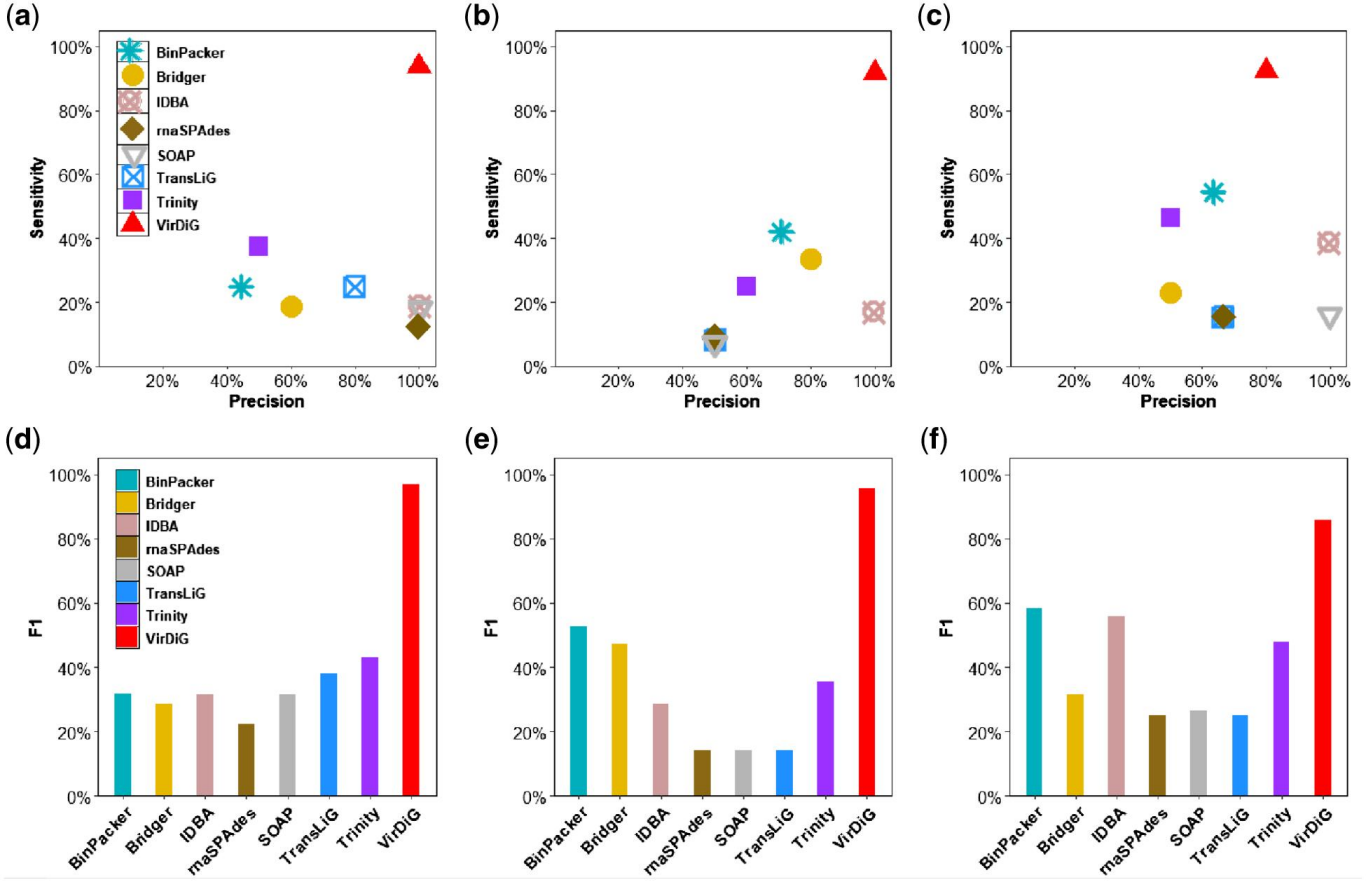
Comparison of precision, sensitivity and F1-score of assemblers on simulated datasets of (a and d) SARS-CoV-1, (b and e) MERS-CoV, and (c and f) SARS-CoV-2.

We conducted tests on VirDiG along with other state-of-the-art *de novo* assemblers using three datasets simulated from different types of coronaviruses. Figure 3 shows the precision, sensitivity, and F1-scores of assemblers on these simulated datasets.

As shown in Fig. 3, VirDiG achieved the highest sensitivity across all simulated datasets, successfully recovering 93.75%, 91.67%, and 92.31% of the full-length reconstructed transcripts for SARS-CoV-1, MERS-CoV, and SARS-CoV-2, respectively. In contrast, the second-ranked assembler, BinPacker, only managed to recover 25%, 41.67%, and 53.85% of full-length reconstructed transcripts. Similarly, Trinity’s results were 37.5%, 25%, and 46.15%. This indicates that VirDiG outperformed BinPacker by recovering an additional 68.75%, 50%, and 38.46% of the full-length reconstructed transcripts, and exceeded Trinity by retrieving an additional 56.25%, 66.67%, and 46.16% of the transcripts. The excellent performance on various datasets underscores the robustness and effectiveness of VirDiG algorithm.

VirDiG also holds a competitive performance in terms of precision, attaining a perfect precision rate of 100% on the simulated datasets for SARS-CoV-1 and MERS-CoV. Although its accuracy on the SARS-CoV-2 dataset was only 80%, lower than that of IDBA-Tran and SOAPdenovo-trans, VirDiG succeeded in reconstructing 12 full-length transcripts, whereas IDBA-Tran and SOAPdenovo-trans only generated 5 and 2 transcripts, respectively.

VirDiG continued to demonstrate its superior performance in terms of F1-score across all simulated datasets. VirDiG achieved F1-scores of 96.77%, 95.65%, and 85.71% on these simulated datasets, respectively. When comparing VirDiG to other methods, it showed a significant performance advantage. For instance, on the SARS-CoV-1 simulated dataset, VirDiG outperformed other methods by a substantial margin of at least 53.91%. Similarly, on the simulated dataset of MERS-CoV, VirDiG surpassed other methods by at least 43.02%. Despite BinPacker attained a relatively high F1-score (58.33%) compared to other traditional assemblers on the SARS-CoV-2 dataset, it still fell short of VirDiG by a margin of 27.38%. These results highlight the superior performance of VirDiG in assembling full-length transcripts across the datasets analysed.

### 3.3 Evaluation on real datasets

**Figure 4.**
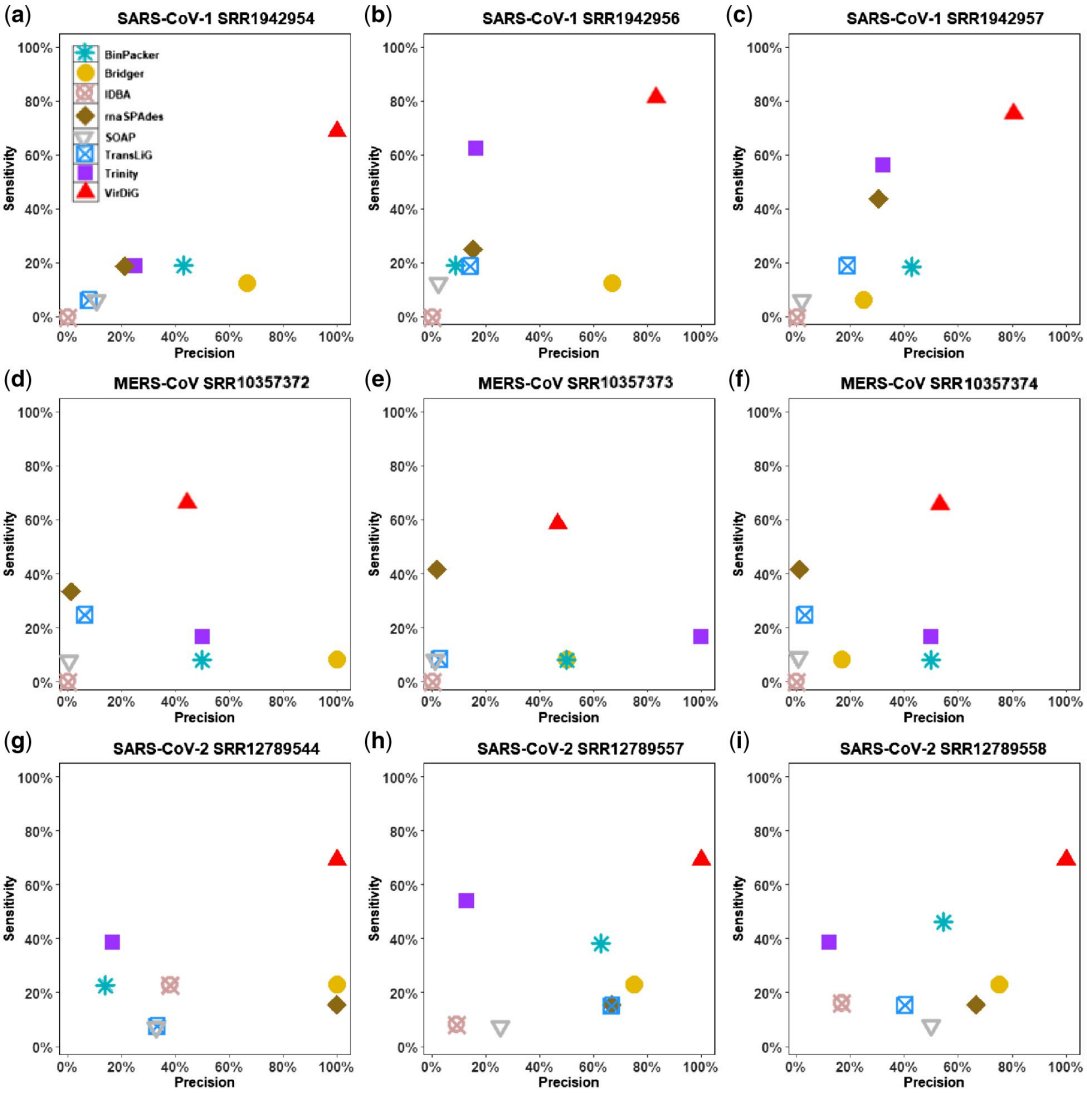
Comparison of the precision and sensitivity distributions of *de novo* transcriptome assemblers across nine real RNA-Seq datasets: (a–c) SARS-CoV-1; (d–f) MERS-CoV; (g–i) SARS-CoV-2.

In this section, we conducted a benchmark of assemblers using 9 real viral datasets from SARS-CoV-1, MERS-CoV, and SARS-CoV-2. This evaluation on actual viral datasets allows us to assess the performance of the assemblers specifically on these important coronaviruses. Figure 4 presents the precision and sensitivity of the assemblers on these datasets, providing a visual representation of their performance. Additionally, the detailed F1-scores for each tool can be found in Table 1, offering further insights into their overall effectiveness in handling the SARS-CoV-1, MERS-CoV, and SARS-CoV-2 datasets.

**Table 1.** F1-scores achieved by *de novo* assemblers on nine real RNA-Seq datasets.

Dataset	Accession No.	Trinity	IDBA	SOAP	Bridger	BinPacker	TransLiG	rnaSPAdes	VirDiG
SARS-CoV-1	SRR1942954	21.43%	0	8%	21.05%	26.09%	7.14%	20%	**81.48%**
	SRR1942956	25.64%	0	4.35%	21.05%	12%	16.22%	19.05%	**81.25%**
	SRR1942957	40.91%	0	3.13%	10%	26.09%	18.75%	35.9%	**77.42%**
MERS-CoV	SRR10357372	25%	0	0.99%	15.38%	14.29%	9.68%	2.45%	**53.33%**
	SRR10357373	28.57%	0	1.13%	14.29%	14.29%	3.7%	3.7%	**51.85%**
	SRR10357374	25%	0	0.76%	11.11%	14.29%	5.41%	2.4%	**59.26%**
SARS-CoV-2	SRR12789544	23.26%	28.57%	12.5%	37.5%	17.65%	12.5%	26.67%	**81.82%**
	SRR12789557	20.9%	8.33%	11.76%	35.29%	47.62%	25%	25%	**81.82%**
	SRR12789558	18.52%	16%	13.33%	35.29%	50%	22.22%	25%	**81.82%**

Bold values represent the maximum values.

As shown in Fig. 4, VirDiG obtained the highest sensitivity across different datasets. For example, it successfully recovered 68.75%, 81.25%, and 75% of the full-length transcripts on the three datasets of SARS-CoV-1. In comparison, Trinity, the second-best assembler, only managed to recover 18.75%, 62.5%, and 56.25% of these transcripts. Similarly, rnaSPAdes yielded results of 18.75%, 25%, and 43.75%. This means that VirDiG retrieved 50%, 18.75%, and 18.75% more full-length transcripts than Trinity and 50%, 56.25%, and 31.25% more than rnaSPAdes. Moreover, VirDiG’s superiority is further emphasized by its performance on other datasets. It recovered 66.67%, 58.33%, and 66.67% of the full-length transcripts on the three datasets of MERS-CoV and 69.23% on all SARS-CoV-2 datasets, showcasing its robustness and effectiveness across different datasets. The exceptional performance of VirDiG can be attributed to its assembly strategy, which is designed based on the discontinuous transcription mechanism of coronaviruses. In contrast, other methods are developed based on the alternative splicing of eukaryotes. The unique assembly strategy of VirDiG aligns well with the specific characteristics of coronaviruses, leading to its superior performance in recovering full-length reconstructed transcripts.

In terms of precision, VirDiG shows better performance on the SARS-COV-1 and SARS-COV-2 datasets compared to other methods. However, its performance is inferior to algorithms like Trinity and BinPacker on the MERS-CoV datasets. Note that these algorithms assembled a minimal number of full-length reconstructed transcripts on the MERS-CoV datasets. Specifically, Trinity, BinPacker and Bridger suffered from the ‘*Multiple Fragment Fusion Problem*’, recovered an average of 2, 1 and 1 full-length reconstructed transcripts, respectively, whereas, VirDiG obtained an average of 7 full-length reconstructed transcripts on datasets of MERS-CoV. Assemblers such as SOAPdenovo-trans and rnaSPAdes generate a large number of candidate transcripts with only a few closely resembling real transcripts, which further affects their precision.

Table 1 presents the F1-scores achieved by different assemblers on the real datasets. Similar to the previous results, VirDiG outperformed other assemblers with significantly superior F1-scores across all datasets. On average, VirDiG attained an F1-score of 72.23% on real datasets, surpassing other methods by a substantial margin of at least 46.76%. Interestingly, all assemblers (except Trinity) achieved their highest performances on the SARS-CoV-2 datasets. Trinity obtained its best F1-score of 40.91% on the sample SRR1942957 of SARS-CoV-1 datasets, but it still falls short of VirDiG’s performance. The highest F1-score achieved by other methods is 50%, achieved by BinPacker on the SRR12789558 dataset of SARS-CoV-2. In contrast, VirDiG excelled with a F1-score of 81.82% on this dataset, surpassing BinPacker by over 30%. VirDiG shows the smallest advantage over other methods on the SRR10357373 dataset, with a 23.28% advantage over the best-performing Trinity algorithm. We attribute VirDiG’s exceptional performance in F1-score to its high sensitivity and competitive precision.

As shown in the above experiments, VirDiG demonstrates a superior ability to accurately reconstruct coronavirus transcripts. While existing *de novo* transcriptome assembly algorithms perform well in assembling transcripts of eukaryotic organisms, they may not be suitable for coronaviruses. VirDiG can effectively fill the gaps left by these algorithms in handling coronaviruses.

## 4 Discussion

Transcriptome assembly of coronaviruses is crucial to understanding the virus’s transcriptome structure, investigating virus variations, and developing antiviral drugs. However, most existing transcriptome assembly algorithms are designed for eukaryotic organisms and rely solely on current methods to assemble the transcriptome of coronaviruses often leads to challenges such as the ‘Multiple Fragment Fusion Problem’.

In this study, we presented a novel *de novo* assembler VirDiG for coronavirus transcriptome assembly using short RNA-seq reads. VirDiG decomposes RNA-seq reads into k-mers for efficient contig assembly and constructs a discontinuous graph for accurate transcript recovery based on vertex weights, read mapping, and the presence of start and stop codons. When compared to state-of-the-art assemblers, VirDiG consistently outperforms them, especially in sensitivity, across all simulated and real datasets.

The superior performance of VirDiG can be attributed to the following key factors. First, VirDiG constructed a more accurate discontinuous graph based on the transcriptional features of coronaviruses, greatly reducing the number of erroneous connections. Second, VirDiG estimated the sequence range of transcripts by leveraging sequence depth and paired-end information. Finally, VirDiG identified the transcript by incorporating information on start and stop codons into the assembly process. This approach effectively mitigates the common issue ‘*Multiple Fragment Fusion Problem*’ that exists in other algorithms. We anticipate that VirDiG will play an important role in discovering new transcripts in coronavirus transcriptome research using RNA-seq data.

## Supplementary Material

vbaf075_Supplementary_Data

## Data Availability

The source code for VirDiG can be found in the GitHub repository at https://github.com/Limh616/VirDiG.git, while the datasets utilized in this project are available at https://github.com/Limh616/data.git.
