# Supplementary material for: VirDiG: a de novo transcriptome assembler for coronavirus
Source: Bioinform Adv. 2025 Apr 8;5(1):vbaf075. doi: 10.1093/bioadv/vbaf075 (PMC12034387; doi:10.1093/bioadv/vbaf075)
Supplement: vbaf075_Supplementary_Data [file vbaf075_supplementary_data.pdf]

Bioinformatics Advances, YYYY, 0–0
doi: 10.1093/Bioinformatics Advances/xxxxx
Advance Access Publication Date: DD Month YYYY
Manuscript Category

Supplementary

VirDiG: a de novo transcriptome assembler for coronavirus

Minghao Li[1], Xiaoyu Guo[1] and Jin Zhao[1,*]

[1]School of Computer Science and Technology, Qingdao University, Ningxia Road, Shandong, China

[*] Corresponding author. E-mail: zhaojin@qdu.edu.cn

Associate Editor: XXXXXXX

1 Construction of discontinuous graph

Step 1. Vertex Labeling. The genomic RNA sequence is designated as a vertex, referred to as vertex v_1.

Step 2. Start Codon Detection. The corresponding sequence of vertex v_1 is systematically scanned to identify all occurrences of the start codon (ATG). The positions of these start codons are considered as potential translation initiation sites, which will guide the subsequent identification of transcript regions.

Step 3. Jump Reads Detecting. Once the 5’ part of a read aligns with the 5’ leader sequence of the genomic RNA and the rest of the read jumps to align with the start codon, the read is called a jump read. For each detected start codon position, we quickly identify the corresponding jump reads by searching for k-mers that contain this start codon.

Step 4. Vertex Splitting and Adding. If a significant number of jump reads are detected at the start codon of a vertex (let's call it v_1), the vertex is split into two parts. The first part is regarded as a new vertex v_2, which represents the region from the 5' end to the position immediately before the start codon, and v_1 is updated as the second part, representing the region from the start codon to the 3' end of the original v_1. A new vertex, v_3, is then added to represent the portion of the beginning of the genomic RNA aligning to the jump reads. Finally, directed edges are added: one from v_2 to v_1 ($v_2 \rightarrow v_1$), and another from v_3 to v_1 ($v_3 \rightarrow v_1$).

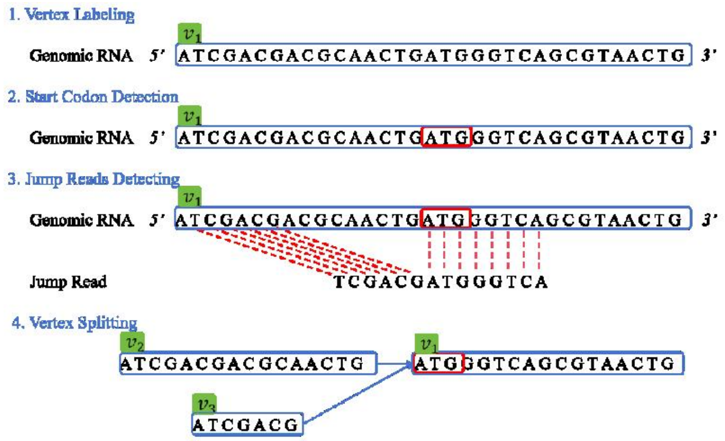

Figure S1. Example of Constructing a Disconnected Graph.

2 Parameter selection

In the algorithm, the parameter (w) represents the importance of paired-reads information in path extension. The parameter (C_{min}) combines abundance information and paired-reads information to establish conditions for path extension. Both parameters are derived from statistical analysis of real data results. The following are the

experimental statistical results of these parameters on the F1-score. While optimal algorithm performance on the MERS-CoV dataset was achieved with multiple parameter pairs, we observed that both excessively large and small parameter values negatively impacted performance. A mid-range parameter pair was selected as the default to ensure optimal algorithm performance (w = 0.7, C_{min}= 1). In laboratory experiments involving the other two viruses (SARS-CoV-1 and SARS-CoV-2), changing the parameter settings within this range did not affect the results of the reconstructed transcripts.

Table S1: Impact of Parameter on Algorithm Performance (SARS-CoV-1 SRR1942954)

w \ C_{min}	0.8	0.9	1	1.1	1.2
0.3	81.48%	81.48%	81.48%	81.48%	81.48%
0.4	81.48%	81.48%	81.48%	81.48%	81.48%
0.5	81.48%	81.48%	81.48%	81.48%	81.48%
0.6	81.48%	81.48%	81.48%	81.48%	81.48%
0.7	81.48%	81.48%	81.48%	81.48%	81.48%
0.8	81.48%	81.48%	81.48%	81.48%	81.48%
0.9	81.48%	81.48%	81.48%	81.48%	81.48%

Table S2: Impact of Parameter on Algorithm Performance (MERS-CoV SRR10357373)

w	0.8	0.9	1	1.1	1.2
0.3	48.00%	41.67%	41.67%	41.67%	41.67%
0.4	51.85%	48.00%	41.67%	41.67%	41.67%
0.5	51.85%	51.85%	48.00%	41.67%	41.67%
0.6	51.85%	51.85%	51.85%	48.00%	48.00%
0.7	50.00%	51.85%	51.85%	51.85%	48.00%
0.8	48.28%	50.00%	51.85%	51.85%	51.85%
0.9	48.28%	48.28%	50.00%	51.85%	51.85%

Table S3: Impact of Parameter on Algorithm Performance (SARS-CoV-2 SRR12789544)

w	0.8	0.9	1	1.1	1.2
0.3	81.82%	81.82%	81.82%	81.82%	81.82%
0.4	81.82%	81.82%	81.82%	81.82%	81.82%
0.5	81.82%	81.82%	81.82%	81.82%	81.82%
0.6	81.82%	81.82%	81.82%	81.82%	81.82%
0.7	81.82%	81.82%	81.82%	81.82%	81.82%
0.8	81.82%	81.82%	81.82%	81.82%	81.82%
0.9	81.82%	81.82%	81.82%	81.82%	81.82%

3 K-mer completeness analysis

We assessed the k-mer completeness of our algorithm and compared it with other de novo assemblers. The sequencing depth of the data we used is typically high, so k-mers with a frequency of 1 may be caused by sequencing errors. To eliminate the impact of sequencing errors on the results, we excluded k-mers with a frequency of 1 when calculating the k-mer completeness and only considered the cumulative frequency of k-mers with a frequency greater than 1. Interestingly, the performance of most algorithms was quite similar. The detailed results are presented in Tables S4-S6.

Table S4. K-mer Completeness Analysis of SARS-CoV-1 Datasets

Assembler	SRR1942954	SRR1942956	SRR1942957
Trinity	95.20%	95.57%	95.20%
IDBA	0.22%	-	-
SOAP	93.96%	93.95%	93.80%
Bridger	88.86%	89.24%	85.31%
BinPacker	95.09%	95.33%	95.12%
TransLiG	95.13%	86.70%	85.65%
rnaSPAdes	93.51%	91.52%	94.25%
VirDiG	94.48%	94.82%	94.54%

Table S5. K-mer Completeness Analysis of MERS-CoV Datasets

Assembler	SRR10357372	SRR10357373	SRR10357374
Trinity	95.76%	95.64%	94.82%
IDBA	1.40%	1.08%	3.40%
SOAP	95.36%	95.22%	94.39%
Bridger	95.65%	95.55%	94.69%
BinPacker	95.65%	95.55%	94.69%
TransLiG	90.27%	95.59%	93.69%
rnaSPAdes	94.49%	95.06%	94.03%
VirDiG	95.65%	95.55%	94.69%

Table S6. K-mer Completeness Analysis of SARS-CoV-2 Datasets

Assembler	SRR12789544	SRR12789547	SRR12789548
Trinity	97.95%	97.15%	97.77%
IDBA	35.90%	26.78%	28.96%
SOAP	96.82%	96.67%	96.57%
Bridger	97.52%	97.50%	97.42%
BinPacker	97.53%	97.79%	97.42%
TransLiG	97.54%	97.39%	97.42%
rnaSPAdes	96.91%	97.73%	97.59%
VirDiG	97.46%	97.42%	97.33%

4 Detailed evaluation results

Transcripts assembled by each algorithm were aligned to the ground truth transcripts using BLAT with 95% sequence identity as cutoff. A ground truth transcript is considered full-length reconstructed if it is covered by an assembled transcript with at least 95% sequence identity and no more than 5% indels. We benchmark the performances of assemblers using sensitivity, precision, and F1-score metrics. Sensitivity is calculated as the ratio of fully reconstructed transcripts to all expressed transcripts in the dataset. Precision is determined by the fraction of full-length reconstructed transcripts among all assembled transcripts. The F1-score takes both sensitivity and accuracy into consideration, which offers a more comprehensive evaluation of the assembly quality. Detailed results are provided in Tables S7-S39.

Table S7. Detailed Evaluation Results for SARS-CoV-1 SRR1942954 Dataset

Accession No.	Assembler	Precision	Sensitivity	F1-scores
**SRR1942954**	Trinity	25.00%	18.75%	21.43%
	IDBA	0.00%	0.00%	0.00%
	SOAP	11.11%	6.25%	8.00%
	Bridger	66.67%	12.50%	21.05%
	BinPacker	42.86%	18.75%	26.09%
	TransLiG	8.33%	6.25%	7.14%
	rnaSPAdes	21.43%	18.75%	20.00%
	VirDiG	**100.00%**	**68.75%**	**81.48%**

Table S8. Detailed Evaluation Results for SARS-CoV-1 SRR1942956 Dataset

Accession No.	Assembler	Precision	Sensitivity	F1-scores
**SRR1942956**	Trinity	16.13%	62.50%	25.64%
	IDBA	0.00%	0.00%	0.00%
	SOAP	2.63%	12.50%	4.35%
	Bridger	66.67%	12.50%	21.05%
	BinPacker	8.82%	18.75%	12.00%
	TransLiG	14.29%	18.75%	16.22%
	rnaSPAdes	15.38%	25.00%	19.05%
	VirDiG	**81.25%**	**81.25%**	**81.25%**

Table S9. Detailed Evaluation Results for SARS-CoV-1 SRR1942957 Dataset

Accession No.	Assembler	Precision	Sensitivity	F1-scores
**SRR1942957**	Trinity	32.14%	56.25%	40.91%
	IDBA	0.00%	0.00%	0.00%
	SOAP	2.08%	6.25%	3.13%
	Bridger	25.00%	6.25%	10.00%
	BinPacker	42.86%	18.75%	26.09%
	TransLiG	18.75%	18.75%	18.75%
	rnaSPAdes	30.43%	43.75%	35.90%
	VirDiG	**80.00%**	**75.00%**	**77.42%**

Table S10. Detailed Evaluation Results for MERS-CoV SRR10357372 Dataset

Accession No.	Assembler	Precision	Sensitivity	F1-scores
**SRR10357372**	Trinity	50.00%	16.67%	25.00%
	IDBA	0.00%	0.00%	0.00%
	SOAP	0.53%	8.33%	0.99%
	Bridger	**100.00%**	8.33%	15.38%
	BinPacker	50.00%	8.33%	14.29%
	TransLiG	6.00%	25.00%	9.68%
	rnaSPAdes	1.27%	33.33%	2.45%
	VirDiG	44.44%	**66.67%**	**53.33%**

Table S11. Detailed Evaluation Results for MERS-CoV SRR10357373 Dataset

Accession No.	Assembler	Precision	Sensitivity	F1-scores
**SRR10357373**	Trinity	**100.00%**	16.67%	28.57%
	IDBA	0.00%	0.00%	0.00%
	SOAP	0.61%	8.33%	1.13%
	Bridger	50.00%	8.33%	14.29%
	BinPacker	50.00%	8.33%	14.29%
	TransLiG	2.38%	8.33%	3.70%
	rnaSPAdes	1.94%	41.67%	3.70%
	VirDiG	46.67%	**58.33%**	**51.85%**

Table S12. Detailed Evaluation Results for MERS-CoV SRR10357374 Dataset

Accession No.	Assembler	Precision	Sensitivity	F1-scores
**SRR10357374**	Trinity	50.00%	16.67%	25.00%
	IDBA	0.00%	0.00%	0.00%
	SOAP	0.40%	8.33%	0.76%
	Bridger	16.67%	8.33%	11.11%
	BinPacker	50.00%	8.33%	14.29%
	TransLiG	3.03%	25.00%	5.41%
	ranSPAdes	1.24%	41.67%	2.40%
	VirDiG	**53.33%**	**66.67%**	**59.26%**

Table S13. Detailed Evaluation Results for SARS-CoV-2 SRR12789544 Dataset

Accession No.	Assembler	Precision	Sensitivity	F1-scores
**SRR12789544**	Trinity	16.67%	38.46%	23.26%
	IDBA	37.50%	23.08%	28.57%
	SOAP	33.33%	7.69%	12.50%
	Bridger	**100.00%**	23.08%	37.50%
	BinPacker	14.29%	23.08%	17.65%
	TransLiG	33.33%	7.69%	12.50%
	rnaSPAdes	**100.00%**	15.38%	26.67%
	VirDiG	**100.00%**	**69.23%**	**81.82%**

Table S14. Detailed Evaluation Results for SARS-CoV-2 SRR12789557 Dataset

Accession No.	Assembler	Precision	Sensitivity	F1-scores
**SRR12789557**	Trinity	12.96%	53.85%	20.90%
	IDBA	9.09%	7.69%	8.33%
	SOAP	25.00%	7.69%	11.76%
	Bridger	75.00%	23.08%	35.29%
	BinPacker	62.50%	38.46%	47.62%
	TransLiG	66.67%	15.38%	25.00%
	rnaSPAdes	66.67%	15.38%	25.00%
	VirDiG	**100.00%**	**69.23%**	**81.82%**

Table S15. Detailed Evaluation Results for SARS-CoV-2 SRR12789558 Dataset.

Accession No.	Assembler	Precision	Sensitivity	F1-scores
**SRR12789558**	Trinity	12.20%	38.46%	18.52%
	IDBA	16.67%	15.38%	16.00%
	SOAP	50.00%	7.69%	13.33%
	Bridger	75.00%	23.08%	35.29%
	BinPacker	54.55%	46.15%	50.00%
	TransLiG	40.00%	15.38%	22.22%
	ranSPAdes	66.67%	15.38%	25.00%
	VirDiG	**100.00%**	**69.23%**	**81.82%**

Table S16. Detailed Evaluation Results of SARS-CoV-2 Simulated Data (20x Real Situation)

Virus	Assembler	Precision	Sensitivity	F1-scores
**SARS-CoV-2**	Trinity	50.00%	38.46%	43.48%
	IDBA	**100.00%**	53.85%	70.00%
	SOAP	75.00%	23.08%	35.29%
	Bridger	25.00%	7.69%	11.76%
	BinPacker	57.14%	30.77%	40.00%
	TransLiG	33.33%	7.69%	12.50%
	rnaSPAdes	66.67%	15.38%	25.00%
	VirDiG	92.86%	**100.00%**	**96.30%**

Table S17. Detailed Evaluation Results of SARS-CoV-2 Simulated Data (30x Real Situation)

Virus	Assembler	Precision	Sensitivity	F1-scores
**SARS-CoV-2**	Trinity	40.00%	30.77%	34.78%
	IDBA	**100.00%**	30.77%	47.06%
	SOAP	87.50%	53.85%	66.67%
	Bridger	40.00%	30.77%	34.78%
	BinPacker	66.67%	46.15%	54.55%
	TransLiG	50.00%	30.77%	38.10%
	rnaSPAdes	**100.00%**	15.38%	26.67%
	VirDiG	80.00%	**92.31%**	**85.71%**

Table S18. Detailed Evaluation Results of SARS-CoV-2 Simulated Data (40x Real Situation)

Virus	Assembler	Precision	Sensitivity	F1-scores
SARS-CoV-2	Trinity	37.50%	23.08%	28.57%
	IDBA	**100.00%**	46.15%	63.16%
	SOAP	**100.00%**	7.69%	14.29%
	Bridger	60.00%	23.08%	33.33%
	BinPacker	62.50%	38.46%	47.62%
	TransLiG	80.00%	30.77%	44.44%
	rnaSPAdes	50.00%	7.69%	13.33%
	VirDiG	92.86%	**100.00%**	**96.30%**

Table S19. Detailed Evaluation Results of SARS-CoV-2 Simulated Data (50x Real Situation)

Virus	Assembler	Precision	Sensitivity	F1-scores
SARS-CoV-2	Trinity	50.00%	46.15%	48.00%
	IDBA	**100.00%**	38.46%	55.56%
	SOAP	**100.00%**	15.38%	26.67%
	Bridger	50.00%	23.08%	31.58%
	BinPacker	63.64%	53.85%	58.33%
	TransLiG	66.67%	15.38%	25.00%
	rnaSPAdes	66.67%	15.38%	25.00%
	VirDiG	80.00%	**92.31%**	**85.71%**

Table S20. Detailed Evaluation Results of SARS-CoV-2 Simulated Data (60x Real Situation)

Virus	Assembler	Precision	Sensitivity	F1-scores
SARS-CoV-2	Trinity	50.00%	46.15%	48.00%
	IDBA	**100.00%**	38.46%	55.56%
	SOAP	**100.00%**	7.69%	14.29%
	Bridger	40.00%	15.38%	22.22%
	BinPacker	57.14%	30.77%	40.00%
	TransLiG	66.67%	15.38%	25.00%
	rnaSPAdes	**100.00%**	15.38%	26.67%
	VirDiG	92.86%	**100.00%**	**96.30%**

Table S21. Detailed Evaluation Results of SARS-CoV-2 Simulated Data (70x Real Situation)

Virus	Assembler	Precision	Sensitivity	F1-scores
SARS-CoV-2	Trinity	50.00%	46.15%	48.00%
	IDBA	66.67%	15.38%	25.00%
	SOAP	**100.00%**	7.69%	14.29%
	Bridger	42.86%	23.08%	30.00%
	BinPacker	54.55%	46.15%	50.00%
	TransLiG	40.00%	15.38%	22.22%
	rnaSPAdes	50.00%	7.69%	13.33%
	VirDiG	92.86%	**100.00%**	**96.30%**

Table S22. Detailed Evaluation Results of SARS-CoV-2 Simulated Data (80x Real Situation)

Virus	Assembler	Precision	Sensitivity	F1-scores
SARS-CoV-2	Trinity	58.33%	53.85%	56.00%
	IDBA	**100.00%**	23.08%	37.50%
	SOAP	**100.00%**	7.69%	14.29%
	Bridger	60.00%	23.08%	33.33%
	BinPacker	62.50%	38.46%	47.62%
	TransLiG	60.00%	23.08%	33.33%
	rnaSPAdes	60.00%	23.08%	33.33%
	VirDiG	92.86%	**100.00%**	**96.30%**

Table S23. Detailed Evaluation Results of SARS-CoV-2 Simulated Data (90x Real Situation)

Virus	Assembler	Precision	Sensitivity	F1-scores
SARS-CoV-2	Trinity	50.00%	46.15%	48.00%
	IDBA	**100.00%**	30.77%	47.06%
	SOAP	**100.00%**	7.69%	14.29%
	Bridger	40.00%	15.38%	22.22%
	BinPacker	50.00%	23.08%	31.58%
	TransLiG	50.00%	23.08%	31.58%
	rnaSPAdes	50.00%	7.69%	13.33%
	VirDiG	92.86%	**100.00%**	**96.30%**

Table S24. Detailed Evaluation Results of SARS-CoV-2 Simulated Data (100x Real Situation)

Virus	Assembler	Precision	Sensitivity	F1-scores
SARS-CoV-2	Trinity	50.00%	46.15%	48.00%
	IDBA	**100.00%**	15.38%	26.67%
	SOAP	**100.00%**	7.69%	14.29%
	Bridger	33.33%	15.38%	21.05%
	BinPacker	44.44%	30.77%	36.36%
	TransLiG	40.00%	15.38%	22.22%
	rnaSPAdes	50.00%	7.69%	13.33%
	VirDiG	92.86%	**100.00%**	**96.30%**

Table S25. Detailed Evaluation Results of SARS-CoV-2 Simulated Data (20x Ideal Situation)

Virus	Assembler	Precision	Sensitivity	F1-scores
SARS-CoV-2	Trinity	**100.00%**	**91.67%**	**95.65%**
	IDBA	**100.00%**	75.00%	85.71%
	SOAP	**100.00%**	**91.67%**	**95.65%**
	Bridger	**100.00%**	75.00%	85.71%
	BinPacker	78.57%	**91.67%**	84.62%
	TransLiG	90.00%	75.00%	81.82%
	rnaSPAdes	**100.00%**	75.00%	85.71%
	VirDiG	81.82%	75.00%	78.26%

Table S26. Detailed Evaluation Results of SARS-CoV-2 Simulated Data (30x Ideal Situation)

Virus	Assembler	Precision	Sensitivity	F1-scores
SARS-CoV-2	Trinity	**100.00%**	91.67%	95.65%
	IDBA	**100.00%**	75.00%	85.71%
	SOAP	**100.00%**	91.67%	95.65%
	Bridger	**100.00%**	75.00%	85.71%
	BinPacker	66.67%	83.33%	74.07%
	TransLiG	**100.00%**	75.00%	85.71%
	rnaSPAdes	**100.00%**	75.00%	85.71%
	VirDiG	**100.00%**	**100.00%**	**100.00%**

Table S27. Detailed Evaluation Results of SARS-CoV-2 Simulated Data (40x Ideal Situation)

Virus	Assembler	Precision	Sensitivity	F1-scores
SARS-CoV-2	Trinity	**100.00%**	**91.67%**	**95.65%**
	IDBA	**100.00%**	75.00%	85.71%
	SOAP	**100.00%**	**91.67%**	**95.65%**
	Bridger	**100.00%**	66.67%	80.00%
	BinPacker	**100.00%**	83.33%	90.91%
	TransLiG	**100.00%**	66.67%	80.00%
	rnaSPAdes	**100.00%**	75.00%	85.71%
	VirDiG	91.67%	**91.67%**	91.67%

Table S28. Detailed Evaluation Results of SARS-CoV-2 Simulated Data (50x Ideal Situation)

Virus	Assembler	Precision	Sensitivity	F1-scores
SARS-CoV-2	Trinity	**100.00%**	**91.67%**	**95.65%**
	IDBA	**100.00%**	75.00%	85.71%
	SOAP	**100.00%**	**91.67%**	**95.65%**
	Bridger	**100.00%**	66.67%	80.00%
	BinPacker	78.57%	**91.67%**	84.62%
	TransLiG	**100.00%**	58.33%	73.68%
	rnaSPAdes	88.89%	66.67%	76.19%
	VirDiG	91.67%	**91.67%**	91.67%

Table S29. Detailed Evaluation Results of SARS-CoV-2 Simulated Data (60x Ideal Situation)

Virus	Assembler	Precision	Sensitivity	F1-scores
SARS-CoV-2	Trinity	**100.00%**	91.67%	95.65%
	IDBA	**100.00%**	75.00%	85.71%
	SOAP	**100.00%**	91.67%	95.65%
	Bridger	**100.00%**	91.67%	95.65%
	BinPacker	78.57%	91.67%	84.62%
	TransLiG	**100.00%**	50.00%	66.67%
	rnaSPAdes	**100.00%**	83.33%	90.91%
	VirDiG	**100.00%**	**100.00%**	**100.00%**

Table S30. Detailed Evaluation Results of SARS-CoV-2 Simulated Data (70x Ideal Situation)

Virus	Assembler	Precision	Sensitivity	F1-scores
SARS-CoV-2	Trinity	**100.00%**	91.67%	95.65%
	IDBA	**100.00%**	75.00%	85.71%
	SOAP	**100.00%**	91.67%	95.65%
	Bridger	**100.00%**	58.33%	73.68%
	BinPacker	85.71%	**100.00%**	92.31%
	TransLiG	**100.00%**	58.33%	73.68%
	rnaSPAdes	**100.00%**	83.33%	90.91%
	VirDiG	**100.00%**	**100.00%**	**100.00%**

Table S31. Detailed Evaluation Results of SARS-CoV-2 Simulated Data (80x Ideal Situation)

Virus	Assembler	Precision	Sensitivity	F1-scores
SARS-CoV-2	Trinity	**100.00%**	91.67%	95.65%
	IDBA	**100.00%**	75.00%	85.71%
	SOAP	**100.00%**	91.67%	95.65%
	Bridger	**100.00%**	83.33%	90.91%
	BinPacker	84.62%	91.67%	88.00%
	TransLiG	90.00%	75.00%	81.82%
	rnaSPAdes	**100.00%**	75.00%	85.71%
	VirDiG	**100.00%**	**100.00%**	**100.00%**

Table S32. Detailed Evaluation Results of SARS-CoV-2 Simulated Data (90x Ideal Situation)

Virus	Assembler	Precision	Sensitivity	F1-scores
SARS-CoV-2	Trinity	**100.00%**	91.67%	95.65%
	IDBA	90.00%	75.00%	81.82%
	SOAP	**100.00%**	91.67%	95.65%
	Bridger	**100.00%**	50.00%	66.67%
	BinPacker	78.57%	91.67%	84.62%
	TransLiG	**100.00%**	50.00%	66.67%
	rnaSPAdes	**100.00%**	75.00%	85.71%
	VirDiG	**100.00%**	**100.00%**	**100.00%**

Table S33. Detailed Evaluation Results of SARS-CoV-2 Simulated Data (100x Ideal Situation)

Virus	Assembler	Precision	Sensitivity	F1-scores
SARS-CoV-2	Trinity	**100.00%**	91.67%	95.65%
	IDBA	**100.00%**	75.00%	85.71%
	SOAP	**100.00%**	91.67%	95.65%
	Bridger	**100.00%**	50.00%	66.67%
	BinPacker	92.31%	**100.00%**	96.00%
	TransLiG	**100.00%**	91.67%	95.65%
	rnaSPAdes	90.00%	75.00%	81.82%
	VirDiG	**100.00%**	**100.00%**	**100.00%**

Table S34. Detailed Evaluation Results of SARS-CoV-1 Simulated Data (50x Real Situation)

Virus	Assembler	Precision	Sensitivity	F1-scores
SARS-CoV-1	Trinity	50.00%	37.50%	42.86%
	IDBA	**100.00%**	18.75%	31.58%
	SOAP	**100.00%**	18.75%	31.58%
	Bridger	60.00%	18.75%	28.57%
	BinPacker	44.44%	25.00%	32.00%
	TransLiG	80.00%	25.00%	38.10%
	rnaSPAdes	**100.00%**	12.50%	22.22%
	VirDiG	**100.00%**	**93.75%**	**96.77%**

Table S35. Detailed Evaluation Results of MERS-CoV Simulated Data (50x Real Situation)

Virus	Assembler	Precision	Sensitivity	F1-scores
MERS-CoV	Trinity	60.00%	25.00%	35.29%
	IDBA	**100.00%**	16.67%	28.57%
	SOAP	50.00%	8.33%	14.29%
	Bridger	80.00%	33.33%	47.06%
	BinPacker	71.43%	41.67%	52.63%
	TransLiG	50.00%	8.33%	14.29%
	rnaSPAdes	50.00%	8.33%	14.29%
	VirDiG	**100.00%**	**91.67%**	**95.65%**

Table S36. Performance of Assemblers in Recovering Non-Canonical Transcripts on Simulated SARS-CoV-1 Dataset.

Virus	Assembler	Precision	Sensitivity	F1-scores
**SARS-CoV-1**	Trinity	20.00%	18.18%	19.05%
	IDBA	**100.00%**	36.36%	53.33%
	SOAP	-	-	-
	Bridger	50.00%	18.18%	26.67%
	BinPacker	60.00%	27.27%	37.50%
	TransLiG	44.44%	18.18%	25.81%
	rnaSPAdes	**100.00%**	9.09%	16.67%
	VirDiG	94.12%	**72.73%**	**82.05%**

Table S37. Performance of Assemblers in Recovering Non-Canonical Transcripts on Simulated MERS-CoV Dataset.

Virus	Assembler	Precision	Sensitivity	F1-scores
**MERS-CoV**	Trinity	54.55%	33.33%	41.38%
	IDBA	**100.00%**	27.78%	43.48%
	SOAP	75.00%	16.67%	27.27%
	Bridger	50.00%	11.11%	18.18%
	BinPacker	66.67%	22.22%	33.33%
	TransLiG	25.00%	11.11%	15.38%
	rnaSPAdes	66.67%	11.11%	19.05%
	VirDiG	91.67%	**61.11%**	**73.33%**

Table S38. Performance of Assemblers in Recovering Non-Canonical Transcripts on Simulated SARS-CoV-2 Dataset.

Virus	Assembler	Precision	Sensitivity	F1-scores
**SARS-CoV-2**	Trinity	27.27%	13.64%	18.18%
	IDBA	**100.00%**	4.55%	8.70%
	SOAP	**100.00%**	9.09%	16.67%
	Bridger	33.33%	13.64%	19.35%
	BinPacker	30.00%	27.27%	28.57%
	TransLiG	21.43%	13.64%	16.67%
	rnaSPAdes	**100.00%**	4.55%	8.70%
	VirDiG	68.00%	**77.27%**	**72.34%**

Table S39. Number of Removed Contigs on the Real Datasets.

Virus	Accession No.	Number of contigs	Number of removed contigs
SARS-CoV-1	SRR1942954	78	59
	SRR1942956	117	88
	SRR1942957	117	97
MERS-CoV	SRR10357372	68	42
	SRR10357373	73	48
	SRR10357374	129	95
SARS-CoV-2	SRR12789544	93	82
	SRR12789557	35	24
	SRR12789558	33	22

5 Impact of transcript abundance on assembly performance

We measured transcript expression levels using FPKM (Fragments Per Kilobase of transcript per Million fragments mapped) and divided transcript abundance into three categories — low, medium, and high — based on the ranges of less than 10,000, between 10,000 and 100,000, and greater than 100,000 in the 9 real datasets, respectively.

As shown in Figure S2, VirDiG consistently identifies a high number of medium-abundance transcripts across all datasets. Although its transcript identification rate is slightly lower than that of rnaSPAdes in certain MERS-CoV datasets, it detected eight transcripts in the SARS-CoV-1 sample SRR1942956. For low-abundance transcripts, VirDiG consistently outperforms the other methods, identifying the highest counts; for example, in the SARS-CoV-1 SRR1942956 sample, it detected four low-abundance transcripts. This highlights VirDiG's ability to capture transcripts with lower expression levels, which is essential for understanding their potential biological functions. While its performance in identifying high-abundance transcripts may not always be the highest in specific SARS-CoV-1 datasets, VirDiG generally shows consistent performance across a range of datasets.

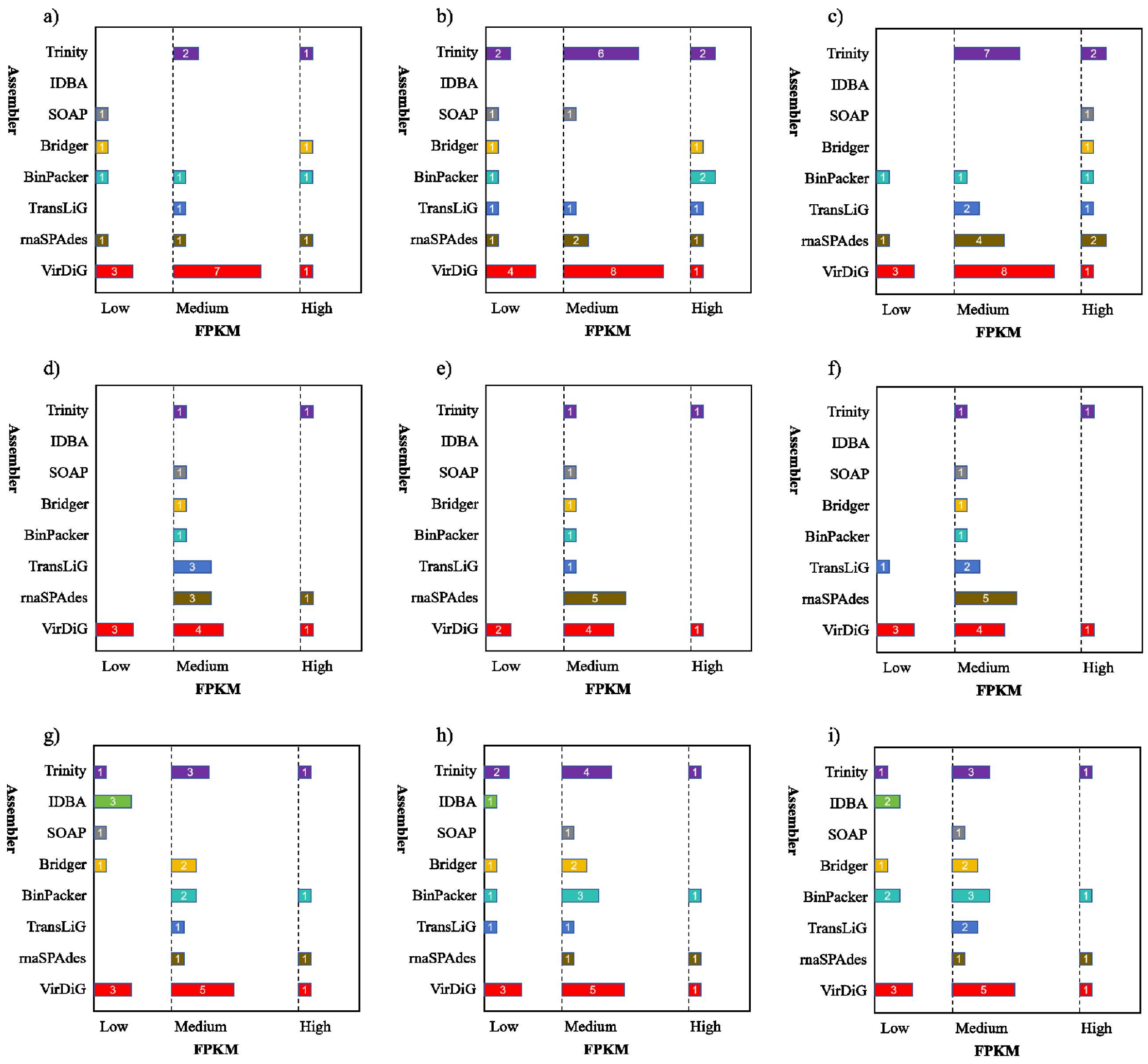

Figure S2. Distribution of Full-length Reconstructed Transcripts Under Different Expression Level : **(a) (b) (c)** SARS-CoV-1; **(d) (e) (f)** MERS-CoV; **(g) (h) (i)** SARS-CoV-2 .

6 A Detailed Comparison of VirDiG and Trinity Algorithm

We examined all the transcripts assembled by Trinity and compared them with those assembled by VirDiG to see if the two tools generate overlapping or unique annotations. The detail results are presented in Figure S3.

As shown in Figure S3, the transcripts assembled by VirDiG and Trinity on the SARS-CoV-1 dataset are mostly the same, but the number of transcripts unique to VirDiG exceeds that of Trinity. On the MERS dataset, the transcripts assembled by VirDiG include all those assembled by Trinity, along with additional transcripts. Furthermore, on the SARS-CoV-1 dataset, the types of transcripts assembled by VirDiG and Trinity differ significantly compared to those on other datasets.

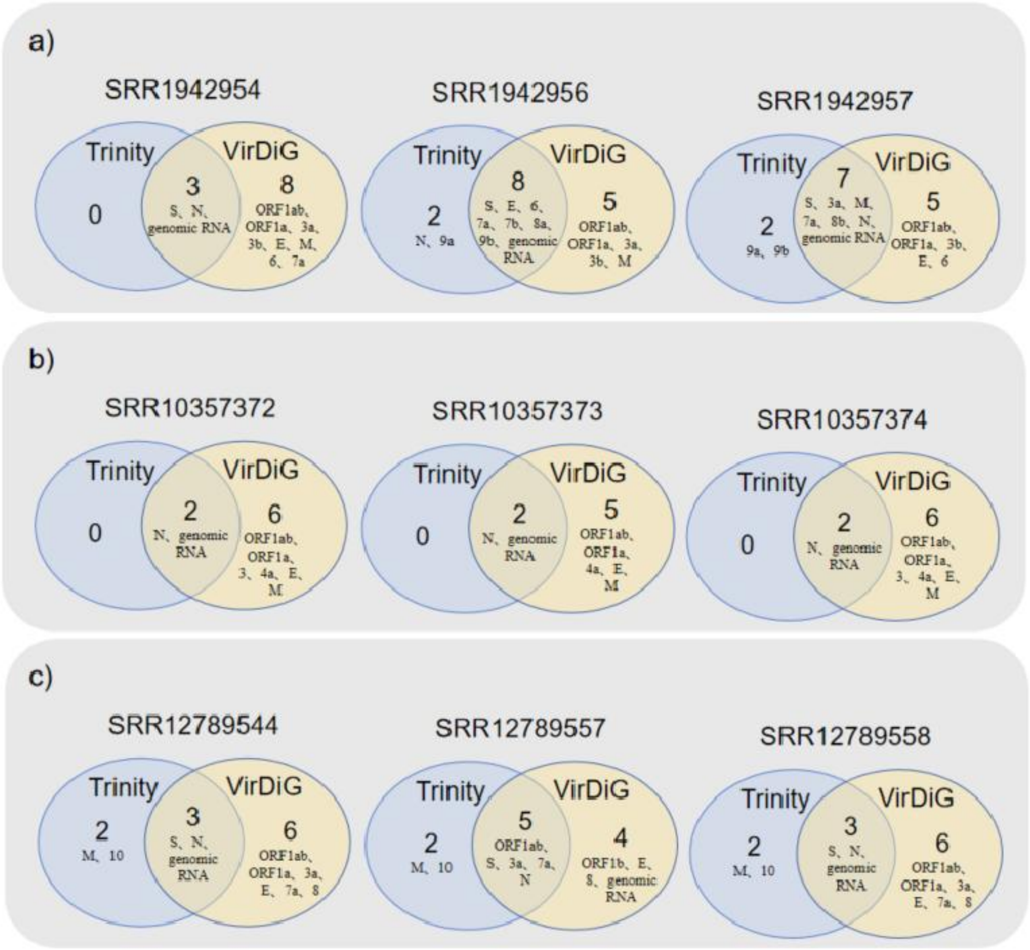

Figure S3. Overlap and Uniqueness of Assembled Transcripts Between Trinity and VirDiG : **(a)** SARS-CoV-1; **(b)** MERS-CoV; **(c)**SARS-CoV-2.

For transcriptome assembly, one of the key indicators of assembly quality is the number of full-length reconstructed transcripts. We first aligned the assembled contigs to the annotated transcripts and then used Trinity's analysis script to check the percentage of the target aligned by the best matching assembled transcripts. An annotated transcript is considered an X%-reconstructed transcript if it has at least X% sequence identity to the best match. Specifically, 95%-reconstructed transcripts are considered full-length. Tables S40-42 show the number of reconstructed transcripts at different identity thresholds and the number of assembled contigs for both Trinity and VirDiG.

Table S40. Number of Transcripts at Different Identity Thresholds on the SARS-CoV-1 Dataset

Dataset	Algorithm	95%	90%	80%	60%	50%	20%	Assembled contigs
SRR1942954	Trinity	3	3	3	3	3	4	12
	VirDiG	11	11	11	11	11	11	11
SRR1942956	Trinity	10	10	10	10	11	12	62
	VirDiG	13	13	13	13	13	13	16
SRR1942957	Trinity	9	9	9	9	9	9	28
	VirDiG	12	12	12	12	12	12	15

Table S41. Number of Transcripts at Different Identity Thresholds on the MERS-CoV Dataset

Dataset	Algorithm	95%	90%	80%	60%	50%	20%	Assembled contigs
SRR10357372	Trinity	2	2	2	2	2	2	4
	VirDiG	8	8	8	8	9	9	18
SRR10357373	Trinity	2	2	2	2	2	2	2
	VirDiG	7	7	7	7	7	9	15
SRR10357374	Trinity	2	2	2	2	2	2	4
	VirDiG	8	8	8	8	8	9	15

Table S42. Number of Transcripts at Different Identity Thresholds on the SARS-CoV-2 Dataset

Dataset	Algorithm	95%	90%	80%	60%	50%	20%	Assembled contigs
SRR12789544	Trinity	5	5	5	5	5	5	30
	VirDiG	9	9	9	9	9	9	9
SRR12789557	Trinity	7	7	7	7	7	8	54
	VirDiG	9	9	9	9	9	9	9
SRR12789558	Trinity	5	5	5	5	6	6	41
	VirDiG	9	9	9	9	9	9	9

As shown in the tables above, VirDiG outperforms Trinity in transcript recognition at most high identity thresholds across all three datasets, showing its stronger assembly capabilities. Trinity struggles at higher identity thresholds but improves when the threshold drops to 60% or lower. Overall, at high identity thresholds (95% and 90%), VirDiG consistently recognizes significantly more transcripts. Even at lower identity thresholds (such as 60%, 50%, and 20%), VirDiG remains stable, demonstrating its adaptability and ability to handle complex data effectively.

7 Evaluation of computing resource usage

De novo assemblers generally consume large computing resources (e.g., CPU time and memory usage). Figure S4 illustrate the CPU time and memory (RAM) usage of each assembler on the real datasets. In various datasets, VirDiG outperforms other assembly tools, showcasing its exceptional capabilities. It consistently achieves shorter processing times across SARS-CoV-1 and SARS-CoV-2 datasets. Additionally, in memory usage, VirDiG maintains good efficiency, showcasing a balanced resource utilization. These qualities position VirDiG as an outstanding choice for data assembly tasks, making it well-suited to meet diverse processing requirements.

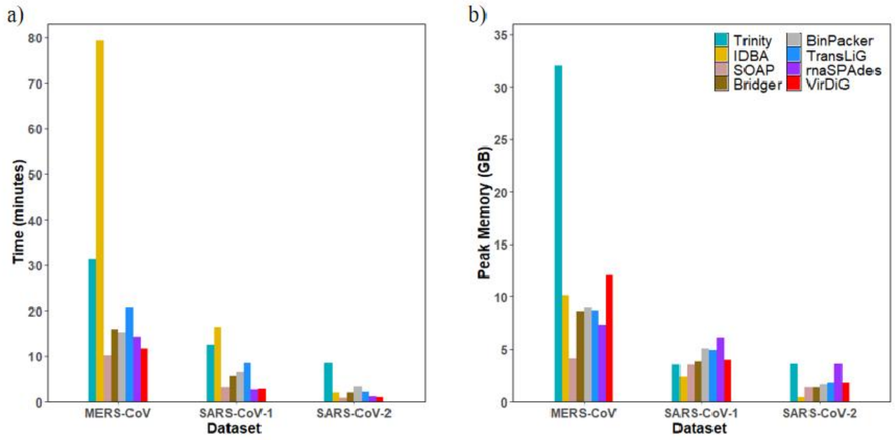

Figure S4. Comparison of CPU Time **(a)** and RAM Usage **(b)** Across Assemblers on Real Datasets.

8 Command lines used for benchmarking

For benchmarking, we used several existing tools, which are listed below. All tools were tested with their default settings unless stated otherwise.

- BLAT: version 2.14.1
  blastn -query transcript.fasta -db reftranscript -out <output> -evalue 1e-20 -dust no -task megablast -num_threads 2 -outfmt 6 -max_target_seqs 3
- Trinity: version 2.15.1
  Trinity --seqType fq --left reads1.fastq --right reads2.fastq --CPU 1 --max_memory 40G --output <output>
- Bridger: version 2014-12-01
  Bridger.pl --seqType fq --left reads1.fastq --right reads2.fastq --CPU 6 -o <output>
- TransLiG: version 1.3
  TransLiG -s fq -p pair -l reads1.fastq -r reads2.fastq -o <output>
- rnaSPAdes: version 3.15.4
  rnaspades.py -1 reads1.fastq -2 reads2.fastq -o <output>
- BinPacker: version 1.0
  BinPacker -s fq -p pair -l reads1.fastq -r reads2.fastq -o <output> -m FR
- IDBA-tran: version 1.1.3
  idba_tran -r read.fa -o <output_dir>
- SOAPdenovo-trans : version 1.0.5
  SOAPdenovo-Trans all -s config_file -o <output>
